# The mediating role of social goals in the relationships between teachers’ transformational leadership, prosocial climate, and motor self-efficacy in physical education students

**DOI:** 10.3389/fpsyg.2025.1695236

**Published:** 2025-09-22

**Authors:** Rafael E. Reigal, Verónica Morales-Sánchez, Rui Matos, Antonio Hernández-Mendo

**Affiliations:** ^1^Departamento Psicología Social, Trabajo Social, Antropología Social y Estudios de Asia Oriental, Universidad de Málaga, Malaga, Spain; ^2^Sport, Exercise and Health Department, Polytechnic University of Leiria, Leiria, Portugal; ^3^Research Center in Sport, Health, and Human Development (CIDESD), Covilhã (pole on the Polytechnic University of Leiria), Leiria, Portugal

**Keywords:** physical education, transformational leadership, prosocial, social goals, motor self-efficacy

## Abstract

**Background:**

There is a limited body of research explaining the associations between transformational leadership and prosocial climate, particularly when considering social goals as mediating variables. Furthermore, no studies have explored the mediating effect of social goals and prosocial climate in the relationships between transformational leadership and motor self-efficacy.

**Purpose:**

The aim of this study was to examine the relationships between transformational leadership, social goals, prosocial climate, and the perception of motor self-efficacy. Specifically, this research intends to determine the mediating role of social goals in the relationships between teacher transformational leadership, prosocial climate, and motor self-efficacy in Physical Education students.

**Method:**

The study involved 392 adolescents (52.81% male, *n* = 207; 47.19% female, *n* = 185) aged between 14 and 16 years (*M* = 15.07; SD = 0.78), who were secondary school students in various educational institutions in the city of Málaga (Spain). Data were collected using the Transformational Teaching Questionnaire, the Social Goals in Physical Education Scale, the School Prosocial Climate Questionnaire, and the Motor Self-Efficacy Scale.

**Results:**

The results revealed multiple relationships among the studied variables. Specifically, a mediation effect of social goals was observed between transformational leadership and prosocial climate, as well as a mediation effect of social goals and prosocial climate in the relationship between transformational leadership and motor self-efficacy.

**Conclusion:**

These findings suggest an association between transformational leadership, prosocial climate, and motor self-efficacy, highlighting the importance of social goals as key variables in understanding these relationships.

## Introduction

Schools, particularly through Physical Education (PE), can contribute to the development of active lifestyles in adolescents (Katewongsa et al., 2022). To foster adherence and motivation toward extracurricular physical activity, students’ experiences and development within educational contexts are highly relevant (Rocliffe et al., 2024). Specifically, PE teachers play a fundamental role, as they serve as role models for adolescents, and the interpersonal relationships established in the classroom can shape students’ thoughts and attitudes (Burgueño et al., 2024). Indeed, the teaching style and interaction of teachers are considered key factors in shaping adolescents’ perceptions of the class and their level of engagement in learning. This justifies the interest in exploring the implications of teachers’ behavior on key variables related to the development of physical activity behavior (Santos et al., 2024).

The importance of teachers in students’ well-being and motivation has been examined through theoretical frameworks such as Self-Determination Theory (SDT) and Achievement Goal Theory (AGT) (Franco et al., 2024). For instance, SDT highlights that autonomy-supportive teaching styles contribute to satisfying students’ basic psychological needs, leading to increased engagement, enjoyment, and satisfaction with the task performed (Mossman et al., 2024). Meanwhile, AGT emphasizes that teachers who promote a task-oriented climate enhance student engagement and enjoyment (Milton et al., 2025). In both cases, these theories have proven useful in explaining how adolescents experience greater well-being within educational settings due to teacher-student interactions, further emphasizing the role of teachers in fostering active lifestyles.

However, these theories have primarily focused on students’ internal motivational processes and the motivational climates generated by teachers (Leo et al., 2022; Zheng et al., 2023), placing less emphasis on other aspects of teacher behavior. Considering that teachers can serve as agents of change and inspiration for their students, as well as facilitators of learning processes, fostering a deeper connection with PE, it is necessary to address these processes from alternative theoretical paradigms that can complement the existing ones (Hennig et al., 2024). In this regard, educational leadership models could provide a suitable framework for understanding the impact of teachers on learning processes, with transformational leadership emerging as a particularly relevant paradigm for explaining students’ experiences and well-being in PE classes (Hidayat and Patras, 2024).

Educational leadership is defined as teachers’ ability to guide and influence students toward achieving learning objectives within an optimal environment (Noetel et al., 2023). Specifically, transformational leadership has been characterized as a form of teacher-student interaction that inspires and motivates students to reach their highest level of personal development, strengthening their commitment to learning (Álvarez et al., 2018; Trigueros et al., 2020). This model comprises four dimensions (Bass and Riggio, 2006; Ibrahim et al., 2014): (a) idealized influence, through which the teacher serves as a role model for students and conveys values to them; (b) motivational inspiration, whereby the teacher encourages students to achieve their goals by fostering enthusiasm and optimism; (c) intellectual stimulation, which involves promoting critical and reflective thinking, as well as creativity and innovation; and (d) individualized consideration, whereby the teacher demonstrates empathy and provides tailored support based on students’ specific needs.

Several studies have highlighted that transformational teachers contribute to enhancing the learning experience and developing students’ competencies (Anderson, 2017; Beauchamp et al., 2011, 2013; Sánchez-García et al., 2024). Strategies such as individualized attention, continuous encouragement for improvement, and the transmission of values related to effort and learning have been shown to facilitate the development of personal skills and self-confidence in tackling tasks (Beauchamp et al., 2011; Hernández-Martos et al., 2024; Sánchez-García et al., 2024). Consequently, if students develop greater skills and confidence in engaging in physical activity within the context of PE, their perception of efficacy in this setting will be reinforced (Beauchamp et al., 2011; Sánchez García et al., 2025).

Self-efficacy refers to individuals’ beliefs about their ability to successfully perform a task (Bandura, 1986; Bandura, 1997) and is a crucial psychological variable in effectively approaching a given activity. Specifically, within PE classes, a distinct dimension known as motor self-efficacy is particularly relevant, as it relates to students’ perceived ability to perform motor tasks (Morales Sánchez et al., 2021). Studies such as those by Sánchez-García et al. (2024) have underscored the association between transformational leadership and perceived motor self-efficacy in the context of PE. Although the development of motor self-efficacy is influenced by multiple factors (Bandura, 1997), it is reasonable to assume that effective learning in PE classes, along with students’ recognition of improvements in their motor skills, would strengthen their self-evaluation. This is particularly significant, as enhanced motor self-efficacy is likely to encourage physical activity behaviors and increase students’ commitment to PE classes and other physical activity contexts (Beauchamp et al., 2011; Fraile-García et al., 2019; Noetel et al., 2023).

Secondly, research has explored the associations between teachers’ interaction styles and the promotion of prosocial behaviors in class. Specifically, interaction styles based on autonomy support (Cheon et al., 2019, 2023) or strategies derived from the Sport Education Model (Manninen and Campbell, 2022) have been found to be effective. Prosocial behaviors play a key role in fostering respect, perceived support, empathy, and collaboration during physical activity, all of which can influence students’ learning experiences, well-being, and engagement, as well as their perceived competence (García-González et al., 2023). The collective manifestation of prosocial behaviors perceived by students is referred to as prosocial climate (García-González et al., 2023), which contributes to the development of positive classroom experiences, increased enjoyment, and a greater willingness to engage in learning (Cheon et al., 2023). Among other aspects, if students perceive a more positive prosocial climate in class, they are more likely to experience greater peer support, avoid feelings of criticism or negative judgment, and develop appropriate self-perceptions related to their participation in PE classes, such as their perception of motor self-efficacy (Opstoel et al., 2020).

Finally, while the relationship between transformational leadership and prosocial behavior has not been extensively examined within the context of PE, it has been explored in competitive sports (Tucker et al., 2010; Turnnidge and Côté, 2018). However, considering that transformational leadership fosters personal consideration, collaborative processes, and social support, and based on its documented effects in sports settings, it would be reasonable to expect that transformational teachers in PE could promote prosocial behaviors among their students (García-González et al., 2023; Ibrahim et al., 2014). Specifically, transformational teachers act as role models for their students by conveying the importance of empathy, positive communication, showing interest in others, addressing individual needs, demonstrating respect, and fostering a sense of personal responsibility. These elements would collectively contribute to the establishment of a prosocial classroom climate (Bass and Riggio, 2006; Hoque and Raya, 2023).

### Present study

Although the relationship between transformational leadership and motor self-efficacy has been described in the context of PE (Beauchamp et al., 2011; Sánchez García et al., 2025), there is a lack of literature explaining the associations between this type of leadership and the prosocial climate in these classes. Since other theoretical paradigms have highlighted how interaction styles are linked to students’ prosocial behavior (Cheon et al., 2019, 2023; Manninen and Campbell, 2022), and research in competitive sports contexts has emphasized that transformational leadership can enhance prosocial attitudes and behaviors in athletes (Tucker et al., 2010; Turnnidge and Côté, 2018), it would be interesting to explore the relationships between transformational leadership and prosocial climate in PE (Sánchez García et al., 2025). The relevance of this lies in understanding and proposing alternative teaching strategies to improve the classroom environment and facilitate learning (García-González et al., 2023; Ibrahim et al., 2014).

Furthermore, as peer interactions influence the classroom environment and learning processes (Cheon et al., 2023; Ellinger et al., 2023; Flores-Piñero et al., 2024), it is essential to determine whether the prosocial climate mediates the relationship between transformational leadership and the perception of motor self-efficacy, in addition to the existence of a direct link between the perception of prosocial behaviors in class and motor self-efficacy (Opstoel et al., 2020). One of the primary contributions of this research is to understand the associations between transformational leadership and prosocial climate. Given that previous studies have described how teachers can foster a prosocial climate in other contexts, it is crucial to examine how this occurs in PE. Since it has been highlighted that PE contributes to students’ social development (Opstoel et al., 2020), this study would enhance the existing knowledge of one of the possible pathways through which this process may occur.

The prosocial climate and students’ experiences are determined by the relationships that emerge within the group (Beni et al., 2017). Thus, students’ social interests and intentions may influence the type of social interactions that occur. In achievement contexts such as PE classes, social goals—which refer to the reasons behind a student’s motivation—are particularly relevant to student behavior (Cecchini Estrada et al., 2011). The social goals that have attracted the most interest among researchers in the context of PE are responsibility goals and relationship goals (Guan et al., 2006a,b). Responsibility goals refer to the intention to maintain positive relationships with classmates, while relationship goals are linked to the desire to respect norms and social rules.

Several studies have highlighted the importance of these social goals in PE, emphasizing their impact on students’ learning experiences, persistence, and enjoyment of the subject (Guan et al., 2006a, 2006b). Specifically, it has been noted that high levels of social goals are associated with a greater sense of group belonging and higher satisfaction with peer relationships (Moreno Murcia et al., 2009). Moreover, Moreno Murcia et al. (2007) indicated that responsibility goals were related to a greater predisposition toward learning and were associated with a more positive perception of self-efficacy. Other studies have also reported associations between relationship goals and perceived competence (Moreno Murcia et al., 2009), reinforcing the importance of peer relationships in enhancing motor learning in this subject.

**Figure 1:**
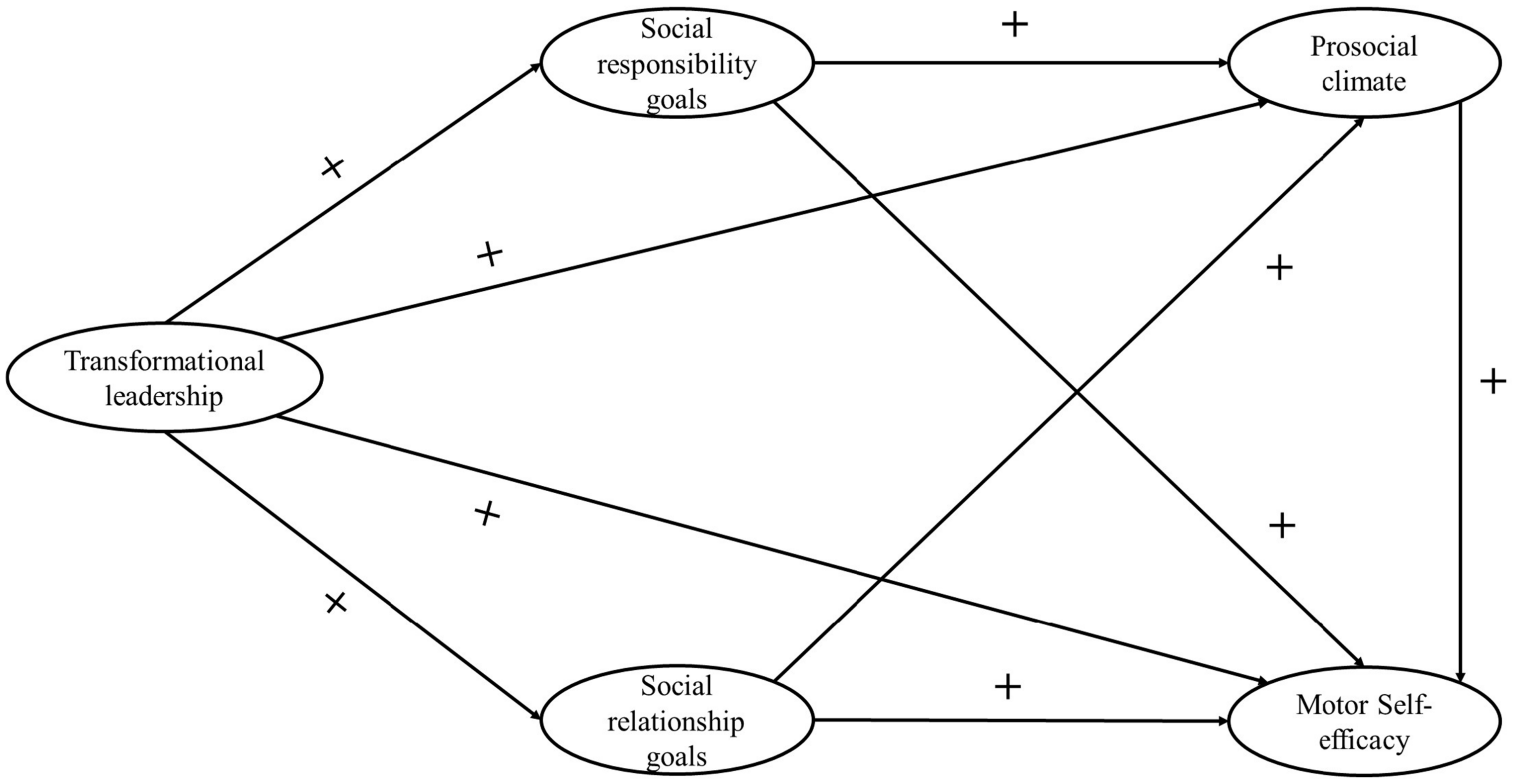
Hypothesized model.

Although social goals have not received as much attention as other theoretical models in explaining students’ experiences in PE, they may play a crucial role in adolescents’ psychosocial development. On the one hand, they can act as a factor that fosters the development of prosocial behavior among students. On the other hand, in the context of this subject, they may contribute to students’ perceptions of competence. Therefore, the aim of this study is to understand the relationships between transformational leadership, social goals, prosocial climate, and motor self-efficacy perception. We hypothesize that (Figure 1): (1) a transformational teaching style will be positively associated with social responsibility goals, social relationship goals, prosocial climate, and motor self-efficacy in PE classes; (2) prosocial climate will be positively related to motor self-efficacy; and (3) social responsibility goals and social relationship goals will mediate the associations between a transformational teaching style, prosocial climate, and motor self-efficacy.

## Methods

### Research design

This study employed an associative, explanatory design (Ato et al., 2013). Specifically, a structural equation model was developed to analyze the associations between variables.

### Participants

Three hundred and ninety-two adolescents (52.81% male, *n* = 207; 47.19% female, *n* = 185) participated in this study. They attended schools in the city of Málaga (Spain), and were aged between 14 and 16 years old (*M* = 15.07; SD = 0.78). All participants attended Physical Education (PE) classes twice a week and followed a similar school curriculum for this subject. Moreover, the socioeconomic characteristics of their school environments were comparable. Additionally, all participants had the same PE teacher during both the previous and the current academic year. To avoid potential biases from other reference figures in competitive sports settings, only students who were not engaged in competitive sports were included. The inclusion criteria were: (a) being enrolled in the 3rd or 4th year of Compulsory Secondary Education; (b) having attended PE classes regularly during the 2023/2024 academic year (>90% of classes); and (c) having been in the same class the previous academic year. Exclusion criteria included: (a) difficulties in reading and understanding the study questions; (b) recent injuries preventing participation in PE; (c) recent enrolment in the school; (d) having had a different PE teacher the previous year; and (e) current engagement in competitive sports, to avoid external leadership influences in physical activity settings.

### Instruments

#### Transformational Teaching Questionnaire (TTQ)

The Spanish version of this questionnaire (Beauchamp et al., 2010) was used to measure the transformational leadership of teachers (Álvarez et al., 2018). It allows the evaluation of the perception that students have about the behaviors of their teachers related to the transformational leadership style. The TTQ begins with the stem “My physical education teacher…”. Consisting of 16 items and four factors (each with four items) to measure individualized consideration (e.g., “Shows that s/he cares about me”), idealized influence (e.g., “Acts as a person that I look up to”), intellectual stimulation (e.g., “Creates lessons that really encourage me to think”), and inspirational motivation (e.g., “Is enthusiastic about what I am capable of achieving”). This is answered on a Likert-type scale from one (never) to five (always). The internal consistency analysis (Cronbach’s alpha) showed values of 0.86 for individualized consideration, 0.83 for idealized influence, 0.84 for intellectual stimulation and 0.89 for motivational inspiration.

#### Social Goals in Physical Education Scale (SGPES)

The Spanish version of this questionnaire (Guan et al., 2006a) was used to measure social goals in physical education (Moreno Murcia et al., 2007). This scale assesses social goals within the context of physical education. It consists of 11 items and two factors: social responsibility goals (five items) (e.g., *“I try to do what the teacher asks me to do”*) and social relationship goals (six items) (e.g., *“I would like to get to know my classmates very well”*). Responses are provided on a Likert-type scale ranging from one (*totally disagree*) to seven (*totally agree*). The internal consistency analysis (Cronbach’s alpha) showed values of 0.84 for social responsibility goals and 0.82 for social relationship goals.

#### School Prosocial Climate Questionnaire (CCPE)

This questionnaire (Roche, 2002) assesses students’ perceptions of the collective prosocial climate within their class group. Consisting of 10 items and one factor (e.g., “We calm those who are nervous and offer encouragement or comfort to those who are feeling sad”). This is answered on a Likert-type scale from one (almost never) to five (almost always). The internal consistency analysis (Cronbach’s alpha) showed values of 0.91.

#### Motor Self-Efficacy Scale (MSES)

Motor Self-Efficacy Scale (MSES) (Hernández Álvarez et al., 2011), which is an adaptation to the motor domain of the Baessler and Schwarzer (1996) General Self-Efficacy Scale (GSE). This instrument analyzes the personal perception of competence to cope with motor tasks. It consists of 10 items and a single factor. The questionnaire was answered using a Likert-type scale from 1 (strongly disagree) to 4 (strongly agree). The internal consistency analysis (Cronbach’s alpha) gave a value of 0.89.

### Procedure

The sample was obtained from high schools in the city of Málaga (Spain). A non-probabilistic (convenience) sampling method was used to select the participants. Twelve high schools, located in districts with a medium-high socioeconomic status, were invited to participate in the research. Five of them agreed to participate. All of them had two or more groups of 25–30 students per year. Firstly, permission was sought from the school to conduct the study, after explaining the research’s purpose. Upon obtaining approval from the school’s administration, families were contacted through a letter to request their children’s participation. Subsequently, informed consent was obtained from the families and the adolescents to participate in the research. In all these cases, participants were informed about the study’s characteristics, the type of data intended to be collected, assured of the confidentiality of their information, and informed of their right to withdraw consent at any time. Throughout the research process, the principles established in the Declaration of Helsinki (World Medical Association, 2013) were respected and approval was obtained from the Ethics Committee of Málaga University to carry out the research. The data were collected during school hours, specifically during Physical Education (PE) classes. Students spent approximately 60 min filling out all the questionnaires. Throughout the information collection process, a researcher was present to address any questions that might arise. Data collection took place between April and May 2024. To minimize potential biases or interferences with other subjects, the questionnaires were administered during Physical Education (PE) classes, ensuring no influence from teachers of other subjects. Additionally, students were explicitly instructed that their responses should refer exclusively to their experiences in PE classes. They were asked to avoid making comparisons or attributions related to other subjects or teachers.

### Data analysis

Means, standard deviations and bivariate correlations were analyzed for all studied variables. For testing the hypothesized model, a two-step maximum likelihood approach (Kline, 2016) was performed in IBM SPSS Amos v.27. Firstly, confirmatory factor analysis (CFA) was performed to analyze the psychometric properties of the proposed model. Composite Reliability (CR) (Raykov, 1997) was evaluated to assess the internal consistency, considering 0.70 as the cut-off value (Hair et al., 2019), while average variance extracted (AVE) was estimated to analyze convergent validity (Hair et al., 2019). Discriminant validity was established when the correlation coefficients were lower than the AVE for each construct exceeding the squared correlations between that construct and any other (Fornell and Larcker, 1981). Secondly, structural equation modeling (SEM) was performed to test the proposed relationships among different constructs. Standardized direct and indirect effects on the variable outcome were analyzed, considering coefficients significant if the 95% Confidence Intervals (CI) did not include zero (Williams and MacKinnon, 2008). The Bootstrap resampling (10,000 samples) considering a bias corrected 95%CI was used to assess the significance of the direct and indirect effects. For CFA and SEM, the following absolute and incremental indices were used for analysis, specifically: Comparative Fit Index (CFI), Tucker-Lewis Index (TLI), Standard Root Mean Residual (SRMR), and Root Mean Square Error of Approximation (RMSEA) with its Confidence Interval (CI: 90%). For these indices, scores of CFI and TLI ≥ 0.90, SRMR and RMSEA ≤ 0.8 were considered as acceptable, following several recommendations (e.g., Byrne, 2016; Hair et al., 2019; Marsh et al., 2004). Note that although the initial model considered the four dimensions of transformational leadership, due to high collinearity and discriminant validity problems among the four factors, a second-order factor was created to represent the general construct of transformational leadership in all subsequent analyses.

### Mediation analysis

Parallel and serial mediation analysis was used to evaluate the relationships among the constructs (Hayes, 2018) using SPSS PROCESS v.3.5 (model 4 – with two parallel mediators, and model 6 – with two serial mediators). Models 4 and 6 allows the control of the indirect effects of each mediator while controlling other variables (i.e., all variables included in the model), also permitting independent mediator effects analysis, and providing regression coefficients for the causal steps of the specified indirect effects. Bootstrap (10,000 samples) was used (Williams and MacKinnon, 2008).

### SEM – multigroup analysis

A Structural Equation Modeling (SEM) multigroup analysis was executed to ensure the proposed model holds equally for boys and girls, as recommended by Byrne (2016). Accordingly, the guidelines put forth by Cheung and Rensvold (2002) and Byrne (2016) were adhered to: (1) the SEM model should exhibit satisfactory fit in each group; (2) adherence to subsequent invariance types, including the unconstrained model, measurement weights, structural weights, measurement intercepts, structural residuals, and measurement residuals. Invariance standards were evaluated by scrutinizing the differences in Comparative Fit Index (ΔCFI<0.01), following the recommendations of Cheung and Rensvold (2002). The analysis was executed using AMOS 28.0.

## Results

### Preliminary analysis

Full Information robust Maximum Likelihood (FIML) was used to manage missing data in the different items (missing at random = 2%) (Enders, 2010). Descriptive statistics, CR coefficients, average variance extracted, and latent correlations are shown in Table 1. Skewness and kurtosis showed normal distribution, since their values were between −2 to +2 and −7 to +7, respectively, (Hair et al., 2019). Also, CR coefficients had a good internal consistency (>0.70). However, the multivariate kurtosis (Mardia’s coefficient) was greater than 5.0 in all cases. Therefore, Bollen-Stine bootstrap on 10,000 samples was employed for further analysis (Nevitt and Hancock, 2001). Regarding latent correlations, all variables showed statistically significant and positive correlations. However, the discriminant validity was not acceptable. Since the correlations between transformational leadership factors were high, the square of the correlations was greater than the AVE in some cases. Additionally, the measurement model tested was incorrect because of the high collinearity of these factors.

**Table 1:** Descriptive statistics, composite reliability coefficients, average variance extracted, and latent correlations of general sample, male and female samples.

Variables	*M*	SD	S	K	CR	AVE	1	2	3	4	5	6	7
Total sample													
1. Individual consideration	4.08	0.87	−0.96	0.44	0.86	0.60	–						
2. Idealized influence	3.77	0.92	−0.71	0.07	0.84	0.57	0.80**	–					
3. Intellectual stimulation	3.71	0.92	−0.54	0.00	0.84	0.57	0.76**	0.79**	–				
4. Motivational inspiration	4.02	0.93	−0.98	0.57	0.89	0.68	0.84**	0.81**	0.77**	–			
5. Social responsibility goals	5.81	0.96	−1.28	1.94	0.84	0.52	0.45**	0.46**	0.40**	0.45**	–		
6. Social relationship goals	5.62	0.96	−1.12	1.65	0.85	0.50	0.34**	0.37**	0.35**	0.35**	0.68**	–	
7. Motor Self-efficacy	3.15	0.54	−0.73	1.05	0.89	0.50	0.40**	0.45**	0.45**	0.46**	0.56**	0.42**	–
8. Prosocial climate	3.49	0.78	−0.26	0.07	0.91	0.51	0.36**	0.39**	0.37**	0.39**	0.61**	0.54**	0.57**
Males													
1. Individual consideration	4.05	0.90	−0.97	0.43	0.87	0.63	–						
2. Idealized influence	3.72	0.91	−0.60	−0.01	0.83	0.56	0.81**	–					
3. Intellectual stimulation	3.66	0.94	−0.44	−0.06	0.84	0.57	0.76**	0.78**	–				
4. Motivational inspiration	3.97	0.96	−1.01	0.73	0.90	0.70	0.82**	0.81**	0.78**	–			
5. Social responsibility goals	5.81	0.92	−1.21	1.98	0.83	0.50	0.45**	0.49**	0.42**	0.49**	–		
6. Social relationship goals	5.67	0.93	−1.11	1.58	0.85	0.51	0.29**	0.32**	0.31**	0.33**	0.65**	–	
7. Motor Self-efficacy	3.19	0.54	−0.72	0.71	0.90	0.49	0.40**	0.48**	0.47**	0.46**	0.60**	0.40**	–
8. Prosocial climate	3.55	0.77	−0.24	0.01	0.91	0.52	0.31**	0.36**	0.34**	0.39**	0.56**	0.48**	0.57**
Females													
1. Individual consideration	4.12	0.85	−0.95	0.43	0.83	0.55	–						
2. Idealized influence	3.81	0.95	−0.84	0.25	0.85	0.60	0.79**	–					
3. Intellectual stimulation	3.77	0.91	−0.66	0.14	0.84	0.57	0.76**	0.80**	–				
4. Motivational inspiration	4.08	0.89	−0.92	0.26	0.88	0.65	0.86**	0.81**	0.76**	–			
5. Social responsibility goals	5.81	1.01	−1.34	1.91	0.85	0.54	0.45**	0.44**	0.38**	0.42**	–		
6. Social relationship goals	5.57	0.99	−1.13	1.72	0.85	0.50	0.40**	0.43**	0.40**	0.38**	0.70**	–	
7. Motor Self-efficacy	3.10	0.53	−0.76	1.55	0.88	0.51	0.42**	0.42**	0.44**	0.47**	0.53**	0.44**	–
8. Prosocial climate	3.44	0.79	−0.27	0.15	0.91	0.50	0.44**	0.44**	0.42**	0.41**	0.66**	0.59**	0.56**

M, mean; SD, standard deviation; S, skewness; K, kurtosis; CR, composite reliability coefficients; AVE, average variance extracted. **p < 0.01.

Therefore, to solve this problem, a second-order factor was calculated from the four factors of the transformational leadership questionnaire. From now on, a measurement model and a structural equation model were developed with this new variable. Table 2 shows the descriptive statistics, CR coefficients, average variance extracted, and latent correlations of the variables after introducing the second order factor. Skewness and kurtosis showed normal distribution. Also, CR coefficients had a good internal consistency (>0.70). Regarding latent correlations, all variables showed statistically significant and positive correlations, except boredom with others which was negative. In addition, considering the squared correlations and AVE scores, all factors demonstrated adequate discriminant validity since the squared correlations of each latent variable were lower than AVE scores in each latent variable. Then, discriminant and convergent validity were acceptable. Therefore, the results indicated that it was appropriate to perform a regression model and analyze the direct and indirect effects between the variables.

**Table 2:** Descriptive statistics, composite reliability coefficients, average variance extracted, and latent correlations after introducing the second order factor of general sample, male and female sample.

Variables	*M*	SD	S	K	CR	AVE	1	2	3	4
Total sample										
1. Transformational leadership	3.89	0.84	−0.78	0.39	0.94	0.79	–			
2. Social responsibility goals	5.81	0.96	−1.28	1.94	0.84	0.51	0.48**	–		
3. Social relationship goals	5.62	0.96	−1.12	1.65	0.85	0.50	0.38**	0.68**	–	
4. Motor Self-efficacy	3.15	0.54	−0.73	1.05	0.89	0.50	0.49**	0.56**	0.42**	–
5. Prosocial climate	3.49	0.78	−0.26	0.07	0.91	0.51	0.40**	0.61**	0.54**	0.57**
Males										
1. Transformational leadership	3.85	0.85	−0.77	0.48	0.94	0.79	–			
2. Social responsibility goals	5.81	0.92	−1.21	1.98	0.83	0.50	0.50**	–		
3. Social relationship goals	5.67	0.93	−1.11	1.58	0.85	0.51	0.34**	0.65**	–	
4. Motor Self-efficacy	3.19	0.54	−0.72	0.71	0.90	0.49	0.49**	0.60**	0.40**	–
5. Prosocial climate	3.55	0.77	−0.24	0.01	0.91	0.52	0.36**	0.57**	0.48**	0.57**
Females										
1. Transformational leadership	3.94	0.82	−0.80	0.30	0.94	0.80	–			
2. Social responsibility goals	5.81	1.01	−1.34	1.91	0.85	0.54	0.46**	–		
3. Social relationship goals	5.57	0.99	−1.13	1.72	0.85	0.50	0.44**	0.70**	–	
4. Motor Self-efficacy	3.10	0.53	−0.76	1.55	0.88	0.51	0.48**	0.53**	0.44**	-
5. Prosocial climate	3.44	0.79	−0.27	0.15	0.91	0.50	0.47**	0.66**	0.59**	0.56**

M, mean; SD, standard deviation; S, skewness; K, kurtosis; CR, composite reliability coefficients; AVE, average variance extracted. **p < 0.01.

### Measurement and structural model

Thus, measurement and structural equations models were generated. The analysis of measurement model included the factors transformational leadership, social responsibility goals, social relationship goals, motor self-efficacy and prosocial climate. Then, the measurement model displayed an acceptable fit to the data: *χ*^2^(547) = 1000.540, *p* < 0.001; CFI = 0.94; TLI = 0.93; SRMR = 0.044; RMSEA = 0.046 90%CI [0.042, 0.051]. Male sample: *χ*^2^(547) = 853.046, *p* < 0.001; CFI = 0.92; TLI = 0.92; SRMR = 0.055; RMSEA = 0.052 90%CI [0.045, 0.059]; female sample: *χ*^2^(547) = 896.263, *p* < 0.001; CFI = 0.91; TLI = 0.90; SRMR = 0.057; RMSEA = 0.059 90%CI [0.052, 0.066]. As we could see, the CR coefficients revealed adequate internal consistency, and the AVE scores showed an acceptable convergent validity (AVE > 0.50). Besides, the structural model displayed an acceptable fit to the data: *χ*^2^(546) = 979.670, *p* < 0.001; CFI = 0.94; TLI = 0.94; SRMR = 0.044; RMSEA = 0.045 90%CI [0.040, 0.050]. Male sample: *χ*^2^(546) = 848.808, *p* < 0.001; CFI = 0.92; TLI = 0.92; SRMR = 0.055; RMSEA = 0.052 90%CI [0.045, 0.059]; female sample: *χ*^2^(546) = 876.980, *p* < 0.001; CFI = 0.91; TLI = 0.90; SRMR = 0.057; RMSEA = 0.057 90%CI [0.050, 0.064]. Therefore, direct and indirect effects were analyzed.

**Figure 2:**
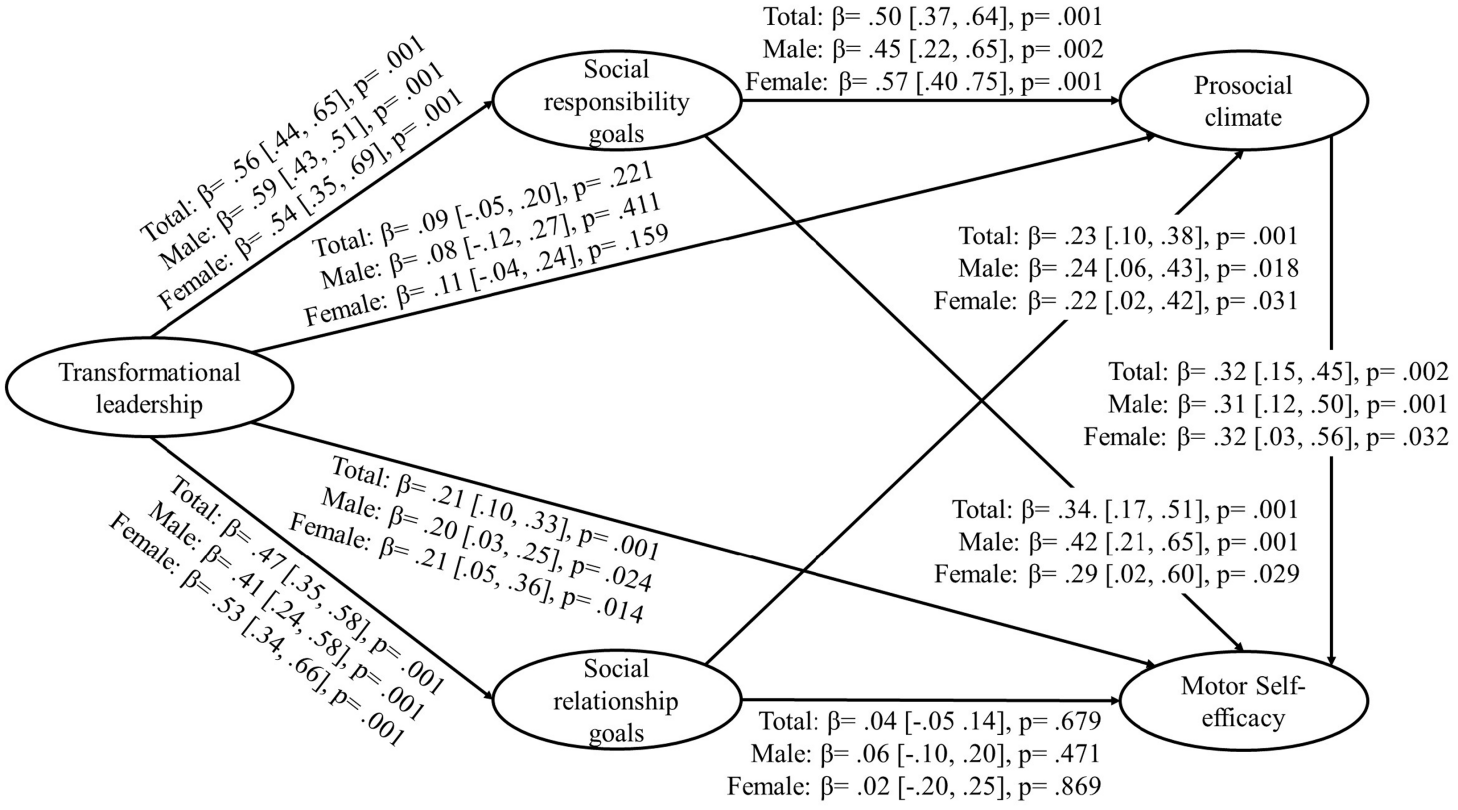
Direct effect coefficients (structural model).

Significant direct effects were found (Figure 2): (a) transformational leadership was positively associated with social responsibility goals, social relationship goals, and motor self-efficacy; (b) social responsibility goals was positively associated with prosocial climate and motor self-efficacy; (c) social relationship goals was positively associated with prosocial climate; (d) prosocial climate was positively associated with motor self-efficacy.

Also, significant indirect effects were found (Table 3): (a) transformational leadership had a positive and indirect effect on prosocial climate via social goals; (b) transformational leadership had a positive and indirect effect on motor self-efficacy via social goals and prosocial climate; (c) social goals had a positive and indirect effect on motor self-efficacy via prosocial climate.

**Table 3:** Direct and indirect effect coefficients.

	*p*	*β*	SE	95%CI	
				LB	UB
Total sample					
TL → Social goals → Prosocial climate	0.001	0.39	0.05	0.30	0.51
TL → Social goals → Prosocial climate → Motor self-efficacy	0.001	0.32	0.05	0.24	0.42
Social responsibility goals → Prosocial climate → Motor self-efficacy	0.001	0.16	0.04	0.08	0.24
Social relationship goals → Prosocial climate → Motor self-efficacy	0.001	0.08	0.03	0.03	0.15
Male					
TL → Social goals → Prosocial climate	0.001	0.36	0.08	0.23	0.55
TL → Social goals → Prosocial climate → Motor self-efficacy	0.001	0.35	0.06	0.26	0.51
Social responsibility goals → Prosocial climate → Motor self-efficacy	0.001	0.14	0.05	0.05	0.26
Social relationship goals → Prosocial climate → Motor self-efficacy	0.021	0.07	0.04	0.01	0.19
Female					
TL → Social goals → Prosocial climate	0.001	0.42	0.08	0.28	0.59
TL → Social goals → Prosocial climate → Motor self-efficacy	0.001	0.31	0.07	0.19	0.48
Social responsibility goals → Prosocial climate → Motor self-efficacy	0.022	0.18	0.08	0.03	0.35
Social relationship goals → Prosocial climate → Motor self-efficacy	0.049	0.07	0.05	0.01	0.20

TL, transformational leadership; β, standardized regression coefficient; SE, standardized error; CI95%, 95% confidence internal; LB, lower bound; UB, upper bound.

### Multigroup analysis

Regarding the multigroup analysis (refer to Table 4), the findings indicate that the proposed SEM model demonstrated invariance across genders, as all the criteria for invariance were met. This implies that factor loadings, structural paths, factor covariances, factor residual variances, and structural and measurement error variances are consistent between genders (∆CFI < 0.01).

**Table 4:** Invariant SEM model between genders.

Model	*χ* ^2^	df	∆*χ*^2^	∆df	*p*	CFI	∆CFI
Male vs. Female							
Unconstrained	1725.824	1,092	–	–	–	0.917	–
Measurement weights	1746.975	1,122	21.151	30	<0.001	0.918	0.001
Structural weights	1753.774	1,131	27.950	39	<0.001	0.918	0.001
Structural covariances	1753.940	1,132	28.116	40	<0.001	0.918	0.001
Structural residuals	1761.294	1,137	35.470	45	<0.001	0.918	0.001
Measurement residuals	1816.232	1,176	90.408	84	<0.001	0.916	0.001

χ^2^ = Chi-square; ∆χ^2^ = differences in value of chi-square; ∆df = differences in degrees of freedom; p = level of significance; CFI = comparative fit index; ∆CFI = differences in the value of the comparative fit index.

### Mediation analysis

**Figure 3:**
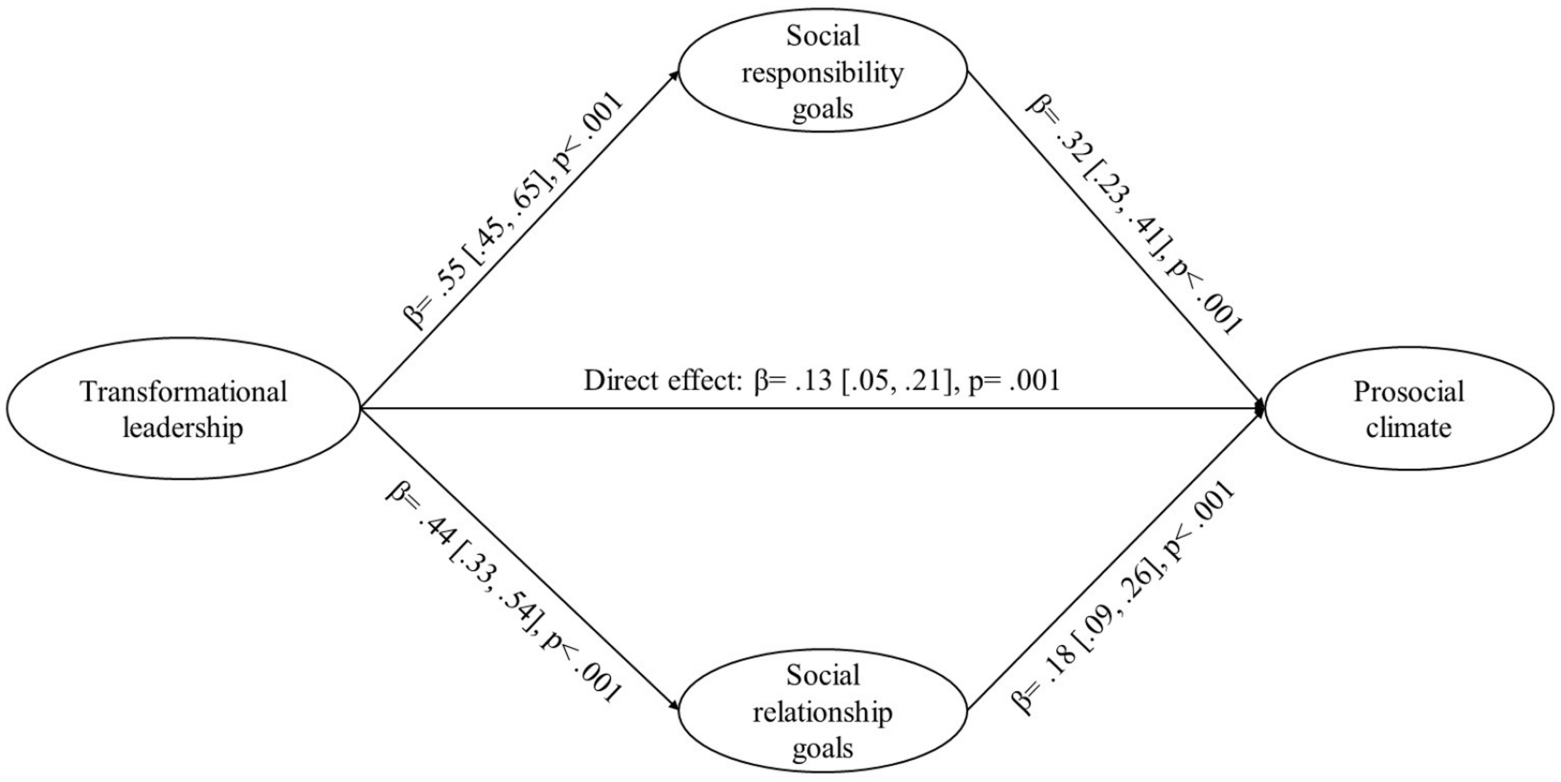
Parallel mediation of social goals in the association between transformational leadership with prosocial climate.

Three mediation analysis were performed. Due to the invariance presented and for better interpretation of the data, these models were performed only for the entire sample. First, a parallel mediation of social goals in the association between transformational leadership with prosocial climate is presented in Figure 3. Results showed a full mediation effect. The model showed a direct effect of *β* = 0.13 [0.05, 0.21] and indirect of *β* = 0.25 [0.19, 0.32]. In addition, the mediation effect via social responsibility goals (*β* = 0.17 [0.12, 0.24]) was greater than via social relationship goals (*β* = 0.08 [0.04, 0.12]), and those differences were statistically significant (*β* = 0.09 [0.02, 0.18]).

**Figure 4:**
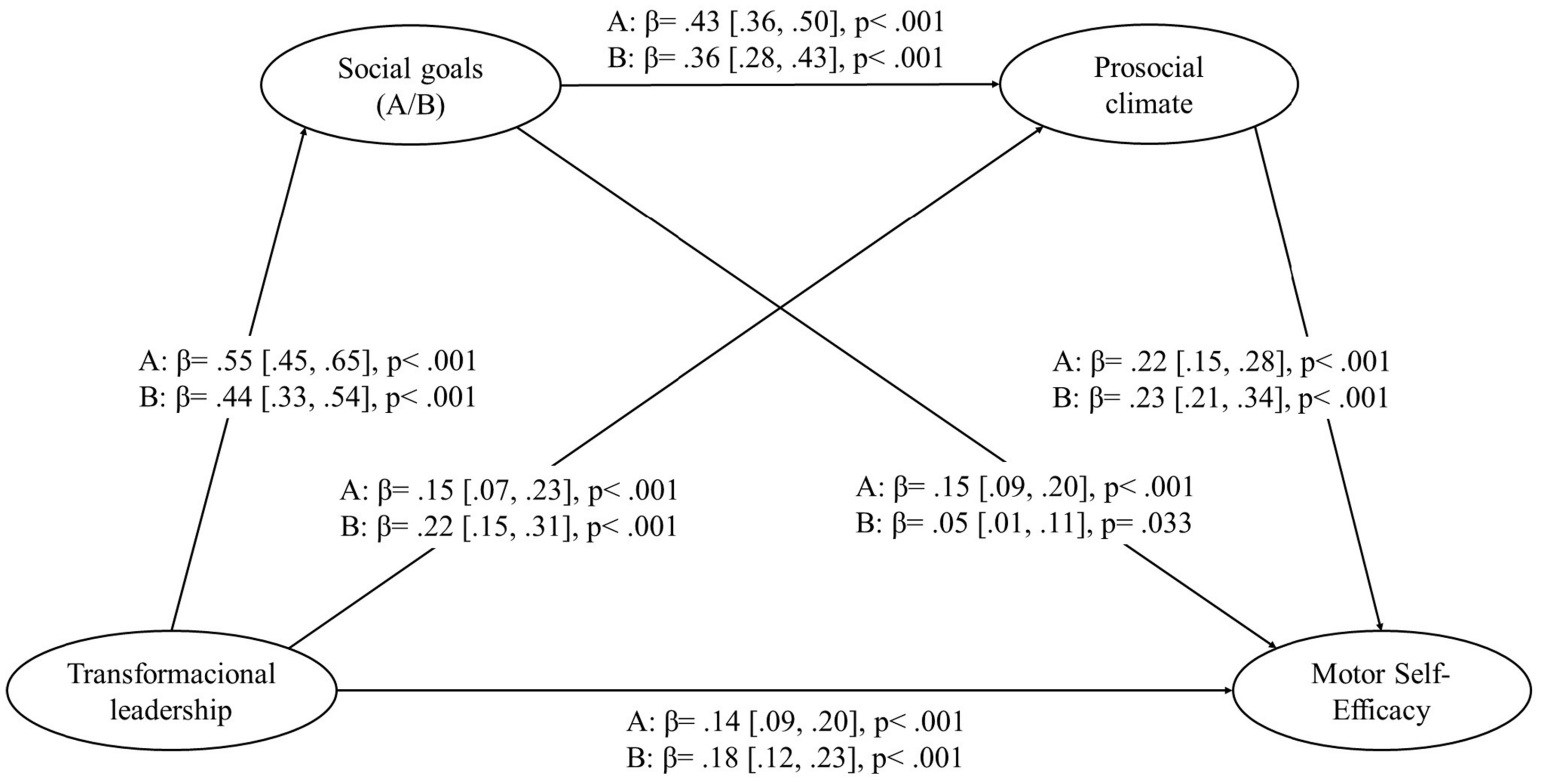
Serial mediation of social goals and prosocial climate in the association between transformational leadership with motor self-efficacy. A = Social responsibility paths; B = Social relationship paths.

Likewise, two serial mediations of social goals and prosocial climate in the association between transformational leadership with motor self-efficacy is presented in Figure 4 and Table 5. For the model that included social responsibility goals, the direct effect was *β* = 0.14 [0.09, 0.20], and the indirect effect was *β* = 0.16 [0.12, 0.21], indicating a full mediation effect. For the model that included social relationship goals, the direct effect was *β* = 0.18 [0.12, 0.23], and the indirect effect was *β* = 0.13 [0.09, 0.18], indicating a partial mediation effect.

**Table 5:** Indirect effect coefficients.

	*β*	SE	95%CI	
			LB	UB
(a) TL → Social responsibility goal → Motor self-efficacy	0.08	0.02	0.04	0.12
(b) TL → Prosocial climate → Motor self-efficacy	0.03	0.01	0.01	0.06
(c) TL → Social responsibility goal → Prosocial climate → Motor self-efficacy	0.05	0.01	0.03	0.08
(a) Minus (b)	0.05	0.03	−0.01	0.10
(a) Minus (c)	0.03	0.03	−0.02	0.08
(b) Minus (c)	−0.02	0.01	−0.05	0.01
(d) TL → Social relationship goal → Motor self-efficacy	0.03	0.01	0.01	0.05
(e) TL → Prosocial climate → Motor self-efficacy	0.06	0.01	0.04	0.09
(f) TL → Social relationship goal → Prosocial climate → Motor self-efficacy	0.04	0.01	0.03	0.07
(d) Minus (e)	−0.04	0.02	−0.08	0.01
(d) Minus (f)	−0.02	0.02	−0.05	0.02
(e) Minus (f)	0.02	0.02	−0.01	0.05

β, standardized regression coefficient; SE, standardized error; CI95%, 95% confidence internal; LB, lower bound; UB, upper bound.

As shown in Table 5, both social goals and the prosocial climate act as significant mediators in explaining the relationships between transformational leadership and motor self-efficacy perception. In the social responsibility goals model, this variable plays a more prominent role as a mediator, while in the social relationship goals model, the prosocial climate has a greater effect.

## Discussion

The primary purpose of the present research was to analyze the relationships between transformational leadership, social goals, prosocial climate, and the perception of motor self-efficacy. We hypothesized that transformational teaching style will be positively associated with social responsibility goals, social relationship goals, prosocial climate and motor self-efficacy in PE class. Also, we considered that prosocial climate will be positively related to motor self-efficacy. In addition, we set that social responsibility goals and social relationship goals would be mediators in the associations between transformational teaching style with prosocial climate and motor self-efficacy. In general, the study variables were positively related, although there were some exceptions that will be discussed later. In addition, a mediation effect of social goals was observed in the relationships between transformational leadership with prosocial climate and motor self-efficacy, which is a relevant finding in this study.

First, correlation analyses and the structural model indicated positive and statistically significant relationships with the social goals of responsibility and relationship. This association is an interesting finding in the context of PE and suggests that this style of interaction could be useful for the development of these goals in students. These relationships could be due to the influence that the transformational teacher would have on the student’s predisposition to be more empathetic, show more interest in others, or increase their individual responsibility (Bass and Riggio, 2006; Hoque and Raya, 2023). In addition, these teachers use strategies that increase support for learning, encourage their students to be more consistent in their efforts, transmit positive values toward daily work, and attend to the needs of each student (Beauchamp et al., 2011; Hernández-Martos et al., 2024; Sánchez-García et al., 2024). Therefore, it would be consistent to consider that an interaction style based on these strategies and behaviors could encourage the stimulation of this type of responsibility and relationship goals.

Besides, scientific literature had previously highlighted how this type of goals was linked to greater student commitment to learning and greater enjoyment in class (Guan et al., 2006a, 2006b; Moreno Murcia et al., 2009). Although there is little evidence in the context of PE, some research agrees with the results of this research that have highlighted the relationship between responsibility goals and a better perception of self-efficacy, as well as the association of relationship goals with a better classroom environment (Moreno Murcia et al., 2007). Furthermore, social responsibility goals have been positively linked to prosocial climate. Among other reasons, it is likely that people who tend to have responsibility goals will be more predisposed to comply with class rules and regulations, accept their peers and respect others, positively influencing the prosocial climate (Guan et al., 2006a; Moreno Murcia et al., 2007, 2009).

In addition, transformational leadership was positively associated with the perception of motor self-efficacy. This is consistent with the influence that transformational leadership theoretically exerts on students, which would have a positive impact on the learning processes and acquisition of skills (Álvarez et al., 2018; Sánchez-García et al., 2024; Trigueros et al., 2020). The strategies used by transformational teachers would contribute to the development of personal skills and the confidence with which they carry out PE classes, which would favor the perception of motor self-efficacy (Beauchamp et al., 2011; Hernández-Martos et al., 2024; Sánchez-García et al., 2024; Sánchez García et al., 2025). Also, a direct relationship between transformational leadership and prosocial behavior has not been observed, which partly contradicts our hypothesis. Although it had not been previously highlighted in PE classes, it was expected given that it had occurred in other contexts of extracurricular physical practice (Tucker et al., 2010; Turnnidge and Côté, 2018). However, an indirect relationship has been produced between these variables through social goals, which indicates that there would be an indirect effect between them and would underline the importance of these variables in the construction of prosocial climates in PE class.

Secondly, relationships were observed between the prosocial climate and the perception of motor self-efficacy, which highlight the importance of classroom environment for the development of students’ perception of competence. As indicated in previous research, and consistent with the results obtained, the perceived prosocial climate could favor the perception of motor competence (García-González et al., 2023; Opstoel et al., 2020). Social interactions are essential for the construction of the perception of efficacy (Bandura, 1986, 1997). Therefore, perceiving a climate of support and respect favors the predisposition to learning and the reinforcement of a positive self-evaluation of the effectiveness of behavior to face the tasks of PE (Cheon et al., 2023). In addition, as will be analyzed below, these relationships will lay the foundations for understanding why transformational leadership indirectly influences the perception of motor self-efficacy, being very relevant to understanding how the teacher’s interactions with students can favor the development of these self-evaluations.

Thirdly, the mediation analyses carried out have indicated the mediation effect that social goals have on the relationship between transformational leadership with the prosocial climate and the perception of motor self-efficacy. A key element in this research is this mediating role, since it allows us to understand how these variables are indirectly related. As we have seen, both social goals have influenced the indirect relationships produced, highlighting the importance of developing this type of goals to improve the social environment of the class, the predisposition to learning and the development of motor self-efficacy (Guan et al., 2006a, 2006b). This would be caused because the development of these social goals would increase the feeling of belonging to the group and improve relationships between peers, favoring the development of prosocial behaviors in the group (Moreno Murcia et al., 2009). Particularly interesting was the fact that that transformational leadership has not been directly related to the prosocial climate in the structural equation model, although it was indirectly related through the social goal of responsibility. Furthermore, in the mediation model, a full mediation effect of social goals was observed. This suggests that the development of social goals thanks to the pedagogical strategies and interactions of the transformational teacher would be contributing to the development of the prosocial climate of the class.

Also, another crucial effect in this research is the absence of a direct relationship between the social goal of relationships and the perception of self-efficacy, but there was an indirect effect through the prosocial climate. In fact, in previous research, the relationships of responsibility goals have been associated more directly with motor self-efficacy, which is consistent with these results, making it necessary to explore indirect pathways through the prosocial climate to find associations with motor self-efficacy (Moreno Murcia et al., 2007, 2009). This, although a partial mediation effect was observed in the relationships between transformational leadership and motor self-efficacy via social goals and prosocial climate, suggests that there is a sequence in the construction of motor self-efficacy from transformational leadership that involves creating an adequate prosocial climate, promoting the acquisition of social goals in students. Therefore, this highlights the role of transformational leadership to explain the influence of teachers on the learning environments in PE (Hidayat and Patras, 2024; Noetel et al., 2023).

This research has some limitations. First, an explanatory design has been used, which is useful for understanding associations between variables, but prevents causal relationships between them to be established. Therefore, it is suggested that longitudinal or quasi-experimental designs be used in a complementary manner to increase knowledge about this phenomenon. Second, this study uses transformational leadership to explain the relationships with social goals, prosocial climate, and motor self-efficacy. However, these types of factors are influenced by other variables that can act as bias, such as experiences in other contexts, use of social networks, parental educational styles, etc. Therefore, it is suggested that other complementary variables be used in future research to observe whether there may be biases that explain the findings of this research. Third, the use of self-report questionnaires can be subject to social desirability biases, especially for measures such as social goals and prosocial climate. Therefore, caution should be exercised when interpreting the results and taking these potential biases into account.

## Conclusion

The results highlighted the positive relationships between the teacher’s transformational leadership, social goals, prosocial climate and motor self-efficacy. Specifically, a mediation effect of social goals was observed between transformational leadership and prosocial climate, as well as a mediation effect of social goals and prosocial climate between transformational leadership and motor self-efficacy. The results highlight the importance of promoting social goals in PE students through a transformational leadership style, to increase the prosocial climate in class and the perception of motor self-efficacy in students.

## Data Availability

The raw data supporting the conclusions of this article will be made available by the authors, without undue reservation.
